# The Enigma of a Traumatic Neuroma: A Case of Nerve Proliferation Without Documented History of Trauma

**DOI:** 10.1002/ccr3.71900

**Published:** 2026-01-22

**Authors:** Shristi Maharjan, Bibek Kattel, Niroj Khanal, Sabin Baniya, Anjani Kumar Yadav, Pradeep Acharya, Ashok Dongol, Mehul Rajesh Jaisani

**Affiliations:** ^1^ Department of Oral and Maxillofacial Surgery BP Koirala Institute of Health Sciences Dharan Nepal; ^2^ College of Dental Surgery BP Koirala Institute of Health Sciences Dharan Nepal; ^3^ Department of Oral and Maxillofacial Surgery Rapti Academy of Health Sciences Dang Nepal

**Keywords:** marginal mandibular nerve, neuroma, oral neuropathy, peripheral nerve sheath tumor

## Abstract

In the differential diagnosis of nerve‐related lesions, even when there is no documented trauma, this case emphasizes the significance of taking traumatic neuroma into account. It also draws attention to the difficulties in determining the cause and the function of surgical excision in treatment.

## Graphical abstract

Intraoperative photograph showing the reflection of full thickness flap over the body of mandible exposing the lesion and specimen following excisional biopsy.

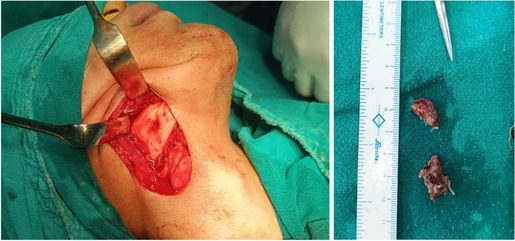

## 1 Introduction

Traumatic neuroma (TN), a rare condition in the oral cavity with the prevalence of 0.3%, usually appears as a solitary nodule close to neurological structures like the mental foramen, tongue, or lips. It results from damage to peripheral nerves brought on by neural damage following tooth extraction, orofacial trauma, and oral surgical procedures. In the attempt at regeneration of nerve, axons proliferate haphazardly, as a result of which the proximal nerve stump is surrounded by connective tissue [1, 2, 3, 4, 5].

This case report highlights the rare occurrence of a traumatic neuroma in the mandibular region, emphasizing its clinical presentation, diagnostic workup, and successful surgical management. It also emphasizes the unusual nature of the condition, as no history of trauma or injury to the region or adjacent tissues was identified, and the patient denied any prior trauma as well.

## 2 Case Presentation

### 2.1 History and Clinical Examination

**FIGURE 1:**
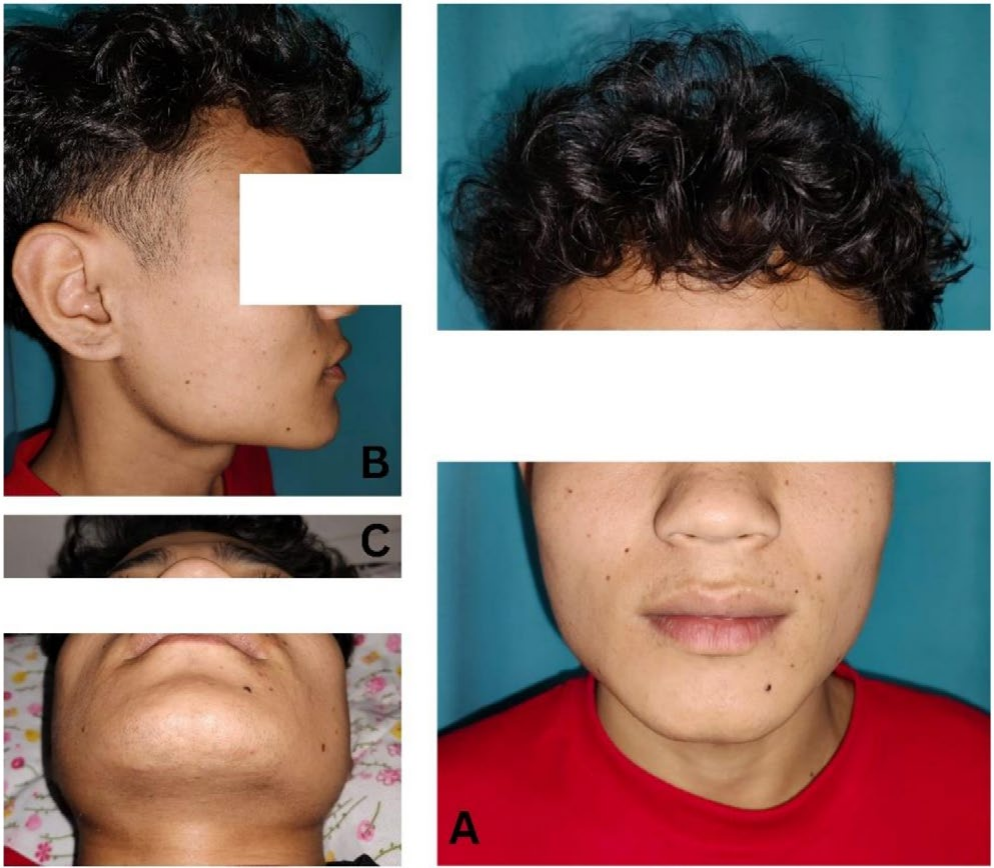
Pre‐treatment extraoral photographs showing small swelling in the right lower posterior region of the jaw. (A) Frontal view, (B) right lateral profile view, and (C) worm's eye view/submento‐vertical view.

A 20‐year‐old male presented to the dental outpatient department (OPD) with a 2‐year history of intermittent pain and a slowly enlarging small swelling in the right lower posterior region of the jaw. The pain was mild, aggravated on touch, and radiated to the anterior and posterior regions of the jaw on the same side. Temporary relief was achieved with ibuprofen. The symptoms were initially present before the extraction of the ipsilateral lower wisdom tooth 2 years ago. Notably, the pain and swelling persisted unchanged following the extraction procedure. Clinical examination revealed a firm, hard swelling approximately 10 × 8 mm in size, located in the lower posterior region of the body of the mandible (Figure 1). The swelling was nonmobile, inferoanterior to the border of the masseter muscle and above the lower border of the mandible. Mouth opening was adequate, and a palpable non‐tender submandibular lymph node was noted. No signs of facial or trigeminal nerve defects were observed, and the patient reported no history of systemic illness or temporomandibular joint dysfunction.

### 2.2 Investigations

**FIGURE 2:**
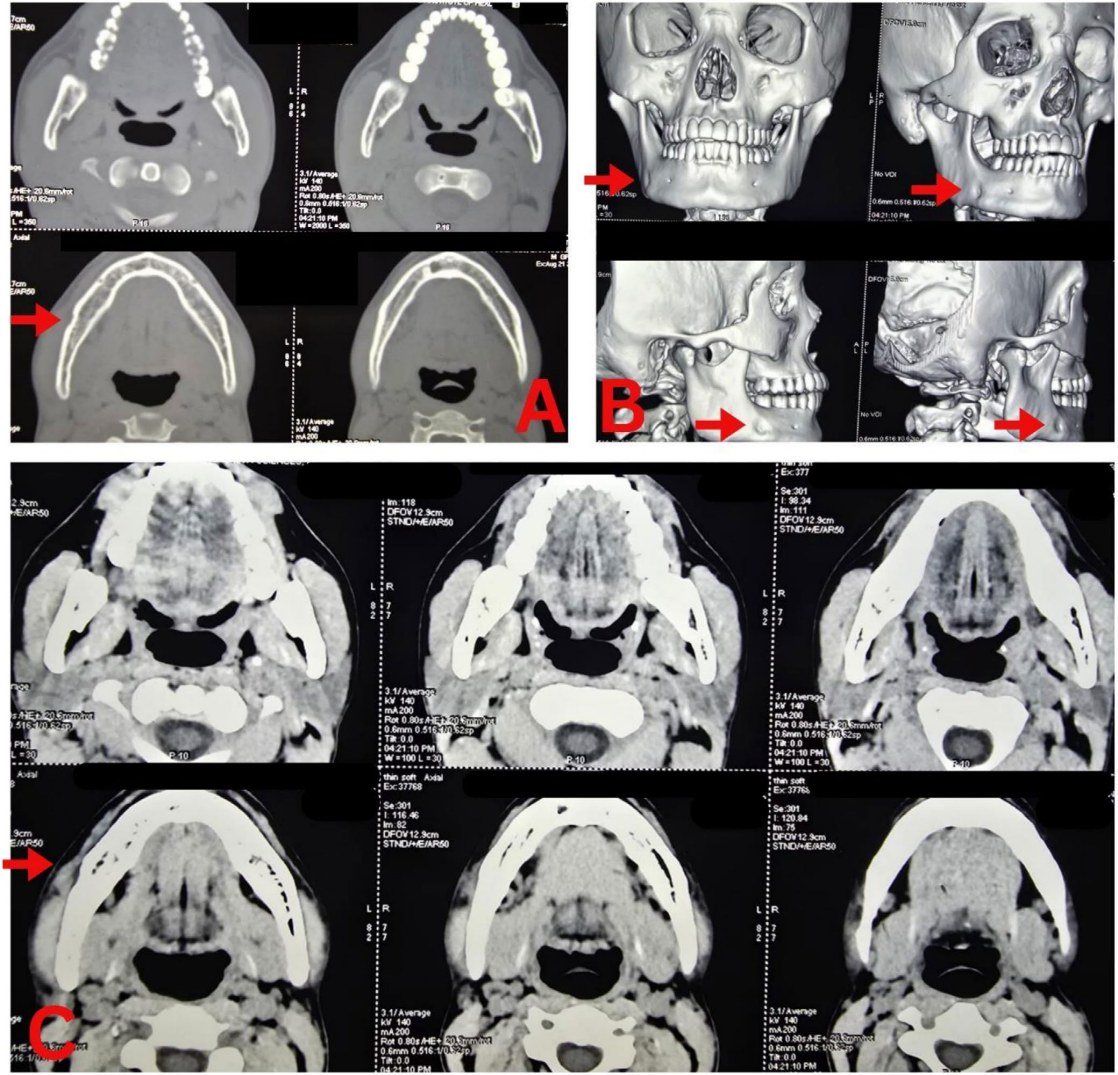
CT images showing (A) bony window, (B) 3D reconstruction showing scalloping of underlying bone due to lesion, (C) soft tissue window (axial section). The area of interest is indicated by a red arrowhead.

Investigations included ultrasonography (USG), which showed a well‐defined nodule measuring 12.8 × 6.6 mm with internal vascularity in the right lower gingivobuccal sulcus. A CT scan revealed a well‐circumscribed, mildly enhancing oval soft tissue density mass in the right masseteric space, causing scalloping of the underlying bone (Figure 2). Prior to the cytological confirmation, the differential diagnoses for the lesion included both neural and salivary gland origin. Fine‐needle aspiration cytology (FNAC) suggested a benign peripheral nerve sheath tumor. Routine laboratory investigations were unremarkable, including a hemoglobin level of 12.5 g/dL, normal leukocyte counts, and negative serological tests for HIV, HBsAg, and HCV.

### 2.3 Treatment

**FIGURE 3:**
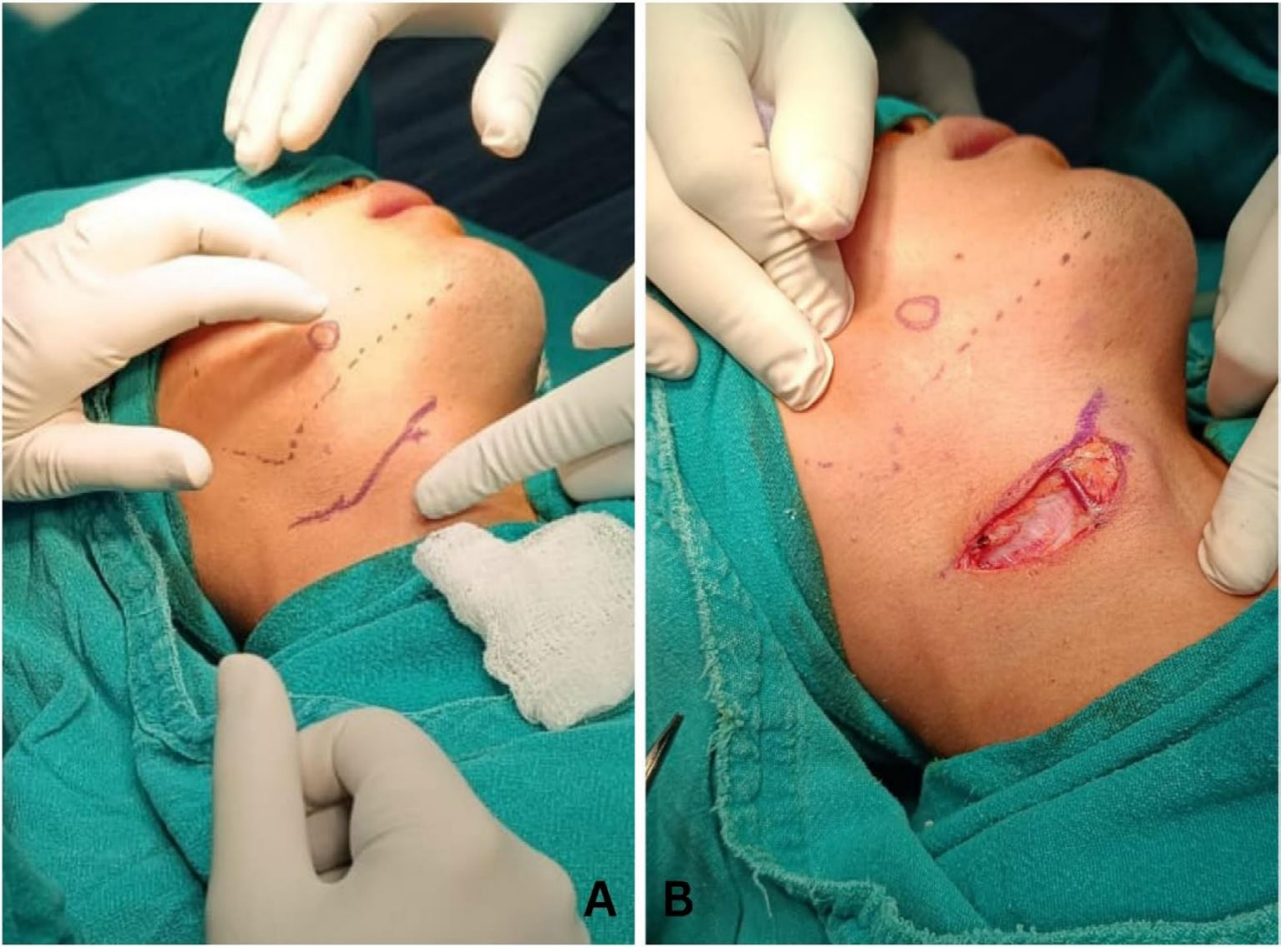
Intraoperative images showing (A) marking of incision line in submandibular region and (b) post‐incision.

**FIGURE 4:**
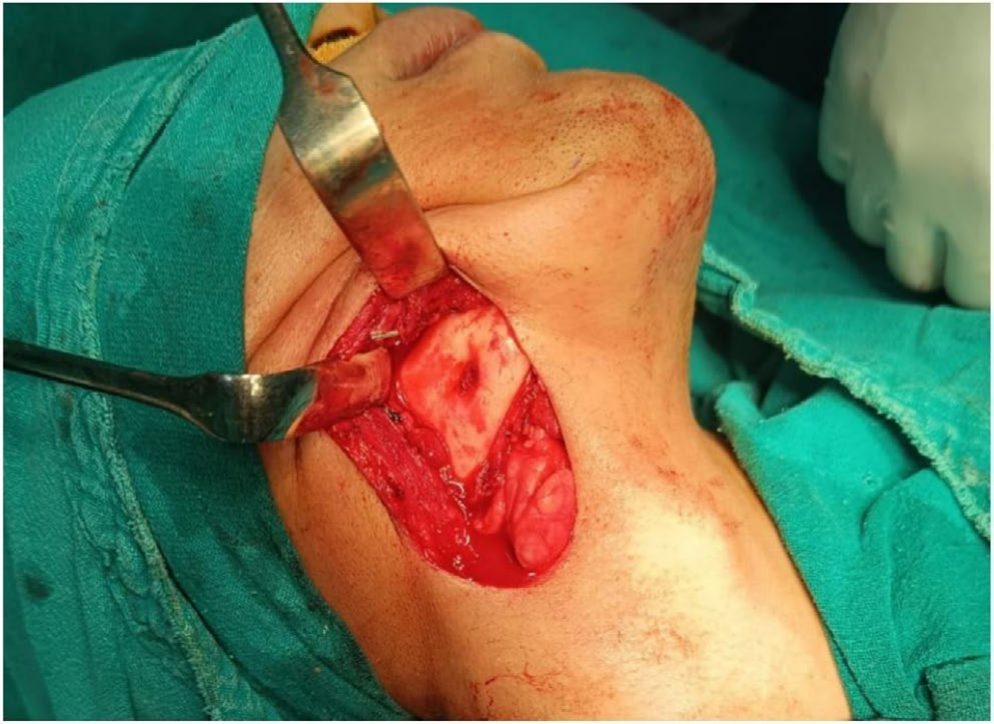
Intraoperative photograph showing the reflection of full thickness flap over the body of mandible exposing the lesion.

**FIGURE 5:**
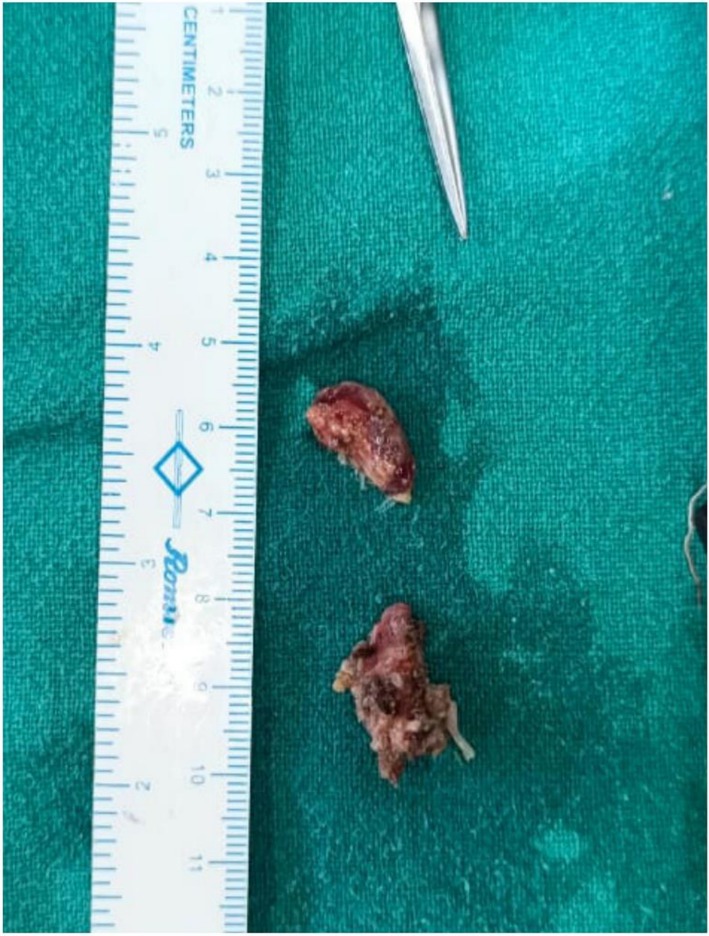
Excised specimen following excisional biopsy. Six soft tissue masses were obtained in total; the image shows the larger tissue chunks submitted for histopathological examination.

The patient underwent an excisional biopsy under general anesthesia after a thorough discussion of potential outcomes and complications, including the possibility of nerve sacrifice. Informed written consent was obtained prior to the procedure. A 7 cm incision (Figure 3) was made in the submandibular region, and submandibular dissection was performed with ligation of head and neck blood vessels. The marginal mandibular nerve was identified and spared. A full‐thickness flap was reflected over the body of mandible to expose the lesion (Figure 4), which was excised using cautery. Closure was done in layers using 3‐0 Vicryl and 4‐0 Prolene sutures. Extubation was uneventful, and the excised specimen (Figure 5) was sent for histopathological analysis.

### 2.4 Gross and Histopathological Examination

**FIGURE 6:**
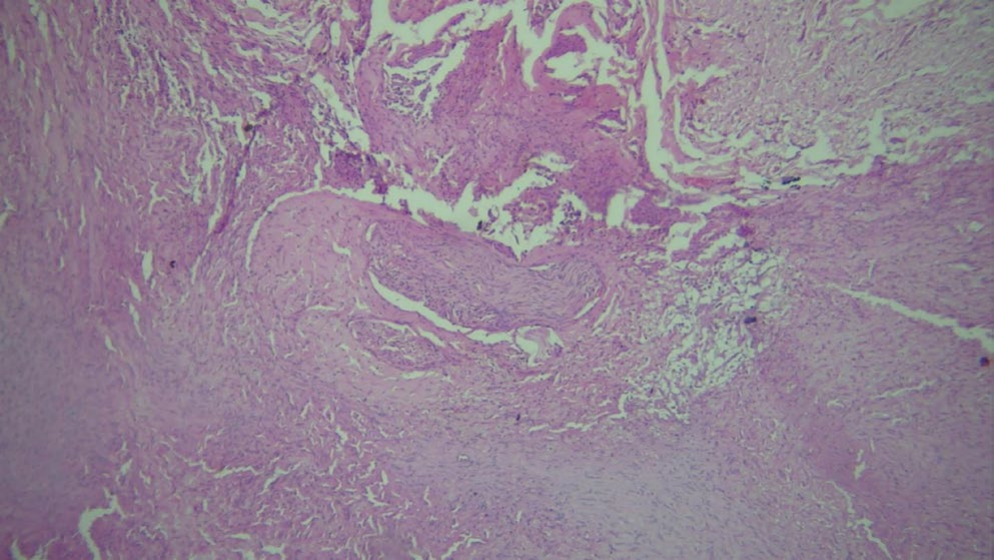
Histopathological image.

Gross examination of the specimen revealed six soft tissue masses from the posterior mandible in relation to tooth 47, white to brown in color, firm in consistency, largest measured 20 × 15 × 10 mm, while another measured 16 × 9 × 7 mm. Histopathological analysis revealed irregularly arranged mature nerve bundles containing spindle cells with wavy nucleus surrounded by dense collagen fibers. The stroma showed endothelial cell‐lined blood vessels of variable sizes with areas of hemorrhage along with bundles of muscle fibers. Separate tissue section shows dense aggregates of lymphocytes (Figure 6). These features were suggestive of traumatic neuroma.

### 2.5 Outcome and Follow Up

**FIGURE 7:**
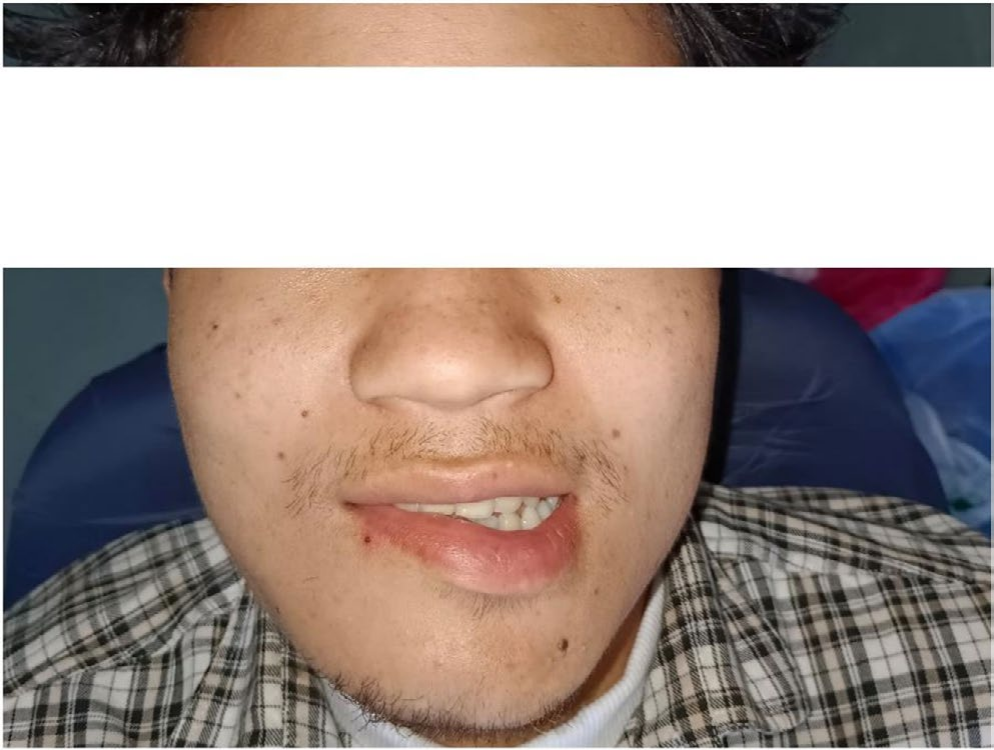
Image showing slight weakness of the right lower lip.

**FIGURE 8:**
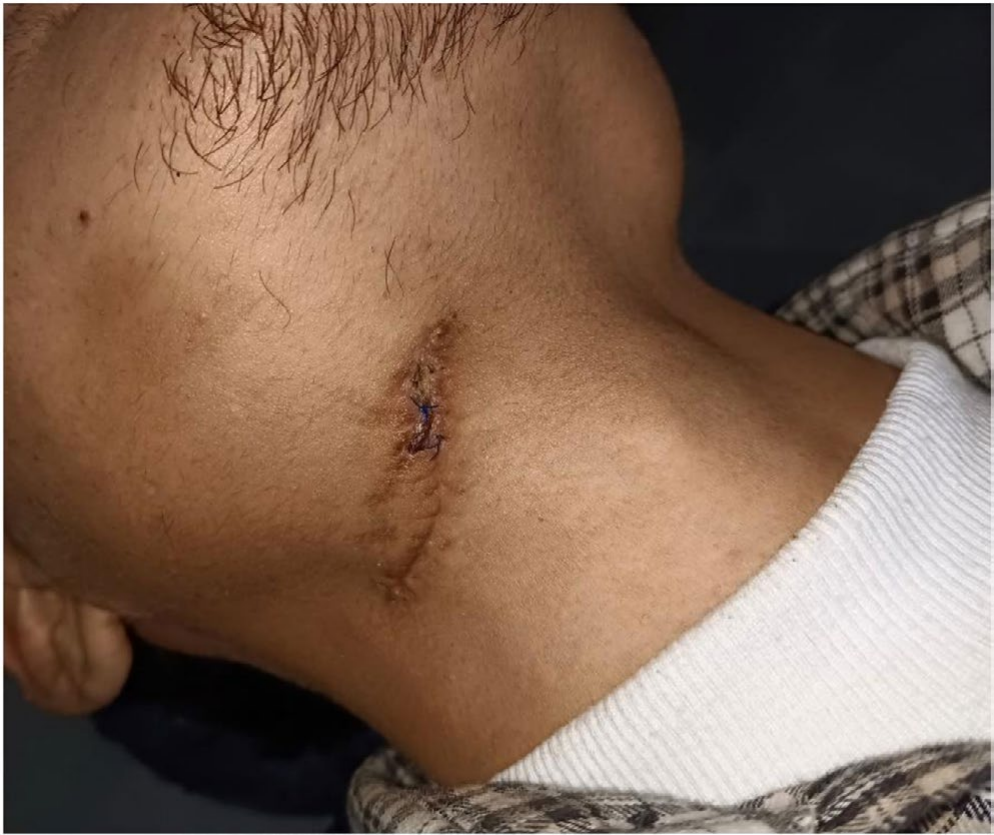
Satisfactory wound healing observed 7 days after discharge. The surgical site shows good epithelialization with no signs of infection or dehiscence.

The patient's hospital course was uneventful, and he was discharged after three postoperative days. At the time of discharge, the patient was clinically and hemodynamically stable with adequate mouth opening, satisfactory wound healing, and slight weakness of the right lower lip (Figure 7). Oral feeding was comfortable. During hospitalization, the patient was treated with antibiotics, analgesics, and proton pump inhibitors. Postoperative IV dexamethasone was used to reduce postoperative edema and compression injury to the nerve. At discharge, medications included Tab Cefixime 200 mg BD PC for 5 days, Tab Ibuprofen TDS PC for 3 days (then SOS), Tab Rabeprazole 20 mg OD AC for 5 days, and Polysporin LA ointment TDS for 5 days. The patient was advised to maintain oral hygiene, ensure adequate oral feeding, and follow up in the dental OPD after 7 days for wound assessment (Figure 8).

This case highlights the diagnosis and management of a rare traumatic neuroma in the mandibular region. Surgical excision was successfully performed, and the patient demonstrated good recovery with no major complications. Continued follow‐up is recommended to monitor nerve function and ensure complete symptom resolution.

## 3 Discussion

Traumatic neuroma is a hyperproliferation of nervous tissue that may cause pain and altered nerve transmission [4]. These lesions can develop anywhere in the body but are rare in the cervicofacial region, with an incidence of approximately 0.34%. Within the oral cavity, they most commonly affect the mental nerve, lower lip, and lingual nerve [2].

A literature review of 31 cases of traumatic neuromas in the oral cavity reported two cases near mandibular molar extraction sites and four cases in the posterior mandible [6]. Similarly, Jones and Franklin reviewed 44,007 oral and maxillofacial pathology specimens from the School of Clinical Dentistry, Sheffield, UK, over 30 years. Their study identified 2452 benign tumors (5.6%), of which 151 (6.2%) were traumatic neuromas [7]. A recent systematic review identified 83 articles focusing on neuromas of the head and neck region, reporting on 386 patients and 393 neuromas. The majority involved the lingual nerve, predominantly following third molar extractions, followed by the cervical plexus, great auricular, and alveolar nerves. Nearly all cases were iatrogenic in origin, highlighting dental and surgical procedures as the principal etiologic factors [8].

Garcia et al. reported a case of a 56‐year‐old patient with hyperesthesia and paresthesia in the lower lip and left third molar region, where a tooth extraction had been performed decades earlier. Radiographic findings showed an osteolytic lesion in the mandibular canal, which was confirmed histologically as a traumatic neuroma. Surgical excision via an intraoral approach led to complete symptom resolution after 3 years [9]. Similarly, Tokuc et al. described an intraosseous traumatic neuroma without any history of trauma, highlighting the need to consider TN in the differential diagnosis of nerve‐related lesions, even in the absence of an obvious history of trauma [10].

Interestingly, in our case, there was no recent history of trauma, except for a tooth extraction more than 2 years ago. However, symptoms were present prior to extraction and persisted afterward. This underscores the need for close follow‐up in cases of tooth extraction near the inferior alveolar nerve, given the potential for TN development.

Clinical presentation may vary. Traumatic neuromas present with variable symptoms, with pain being the most common; either neuralgic or non‐neuralgic, often exacerbated by pressure. Additional sensory disturbances such as paresthesia, anesthesia, or hyperpathia may also occur. Neurologic symptoms result from nerve compression by the tumor, which, owing to its slow growth, allows compensatory mechanisms until a threshold is reached where the nerve is compromised and the symptoms begin [11]. Some authors suggest that disturbances like paresthesia and anesthesia correlate with nerve size [6, 12].

Once a neuroma is formed, surgical removal is indicated, particularly if symptomatic [3]. Conservative measures such as anti‐inflammatory medications, narcotics, and anticonvulsants may provide temporary relief but are often insufficient for long‐term management. Many authors agree that when conservative treatment fails, surgical excision is necessary [13, 14].

In this case, the patient presented with a two‐year history of intermittent pain and a small swelling in the right lower posterior jaw. The pain was mild, aggravated by touch, and radiated to adjacent regions. Fine‐needle aspiration cytology (FNAC) suggested a peripheral nerve sheath tumor. This was a preliminary investigation prior to excision. The definitive diagnosis was made by histopathological evaluation, which is considered the gold standard [15] as TN must be distinguished from ganglioneuroma, ganglioneuroblastoma, subgemmal neurogenous plaques, and neurofibromas. While not performed in this case, immunohistochemical staining like s100 and neurofilament staining plays a crucial role in the diagnosis and subclassification of peripheral nerve sheath tumors and other spindle cell lesions [16].

The distinction between the initial FNAC diagnosis and histopathological diagnosis is based on the difference between viewing individual cells and seeing the tissue architecture. FNAC showed clusters of spindle cells with elongated, wavy, and comma‐shaped nuclei, suggesting benign peripheral nerve sheath tumor. However, it couldn't show the organization of cells within tissue. Histopathology revealed disorganized nerve bundles in a fibrous scar. FNAC correctly identified the lesion's neural origin, whereas only histopathology could reveal the feature that is pathognomonic for a traumatic neuroma.

The patient exhibited drooping of the left lower lip, suggesting the lesion involved the marginal mandibular nerve. Following surgical excision, the patient reported complete symptom resolution, reinforcing the effectiveness of definitive surgical management for traumatic neuromas.

## 4 Conclusion

In conclusion, we present a rare case of traumatic neuroma (TN) with chronic pain that has no documented history of trauma. Its unusual presentation is highlighted by the patient's denial of any past trauma and lack of any discernible trauma or injury to the area. Even in situations where there was not a clear history of trauma, this case highlights the importance of taking TN into account when making a differential diagnosis for nerve‐related lesions.

## Author Contributions

**Shristi Maharjan:** investigation, project administration, software, writing – original draft. **Bibek Kattel:** investigation, project administration, software, writing – original draft. **Niroj Khanal:** investigation, project administration, software, writing – original draft. **Sabin Baniya:** investigation, project administration, software, writing – original draft. **Mehul Rajesh Jaisani:** conceptualization, supervision, validation, visualization, writing – review and editing. **Ashok Dongol:** supervision, writing – review and editing. **Pradeep Acharya:** supervision, writing – review and editing. **Anjani Kumar Yadav:** supervision, writing – review and editing.

## Funding

The authors have nothing to report.

## Ethics Statement

Ethics committee approval was not obtained because we report a clinical case of one patient; we have not included any identifiable information and we obtained written consent for publication from the patient's parents according to the 1964 Helsinki Declaration and its later amendments or comparable ethical standards.

## Consent

Written informed consent was obtained from the patient for the publication of the case report and any accompanying images.

## Conflicts of Interest

The authors declare no conflicts of interest.

## Data Availability

All data underlying the results reported in this case report are included within the manuscript and no additional source data are required.
